# Decoding life's inner workings: advances in quantitative bioimaging

**DOI:** 10.1098/rsob.230329

**Published:** 2023-11-29

**Authors:** Ricardo Henriques, Christophe Leterrier, Aubrey Weigel

**Affiliations:** ^1^ Optical cell biology group, Instituto Gulbenkian de Ciência, Oerias, Portugal; ^2^ MRC Laboratory for Molecular Cell Biology, University College London, London, UK; ^3^ Aix Marseille Université, CNRS, INP UMR7051, NeuroCyto, Marseille, France; ^4^ HHMI Janelia Research Campus, 19700 Helix Drive, Ashburn, VA 20147, USA

**Keywords:** quantitative bioimaging, super-resolution microscopy, single-molecule tracking, machine learning, fluorescence imaging, morphological characterization

## Abstract

This special feature of *Open Biology*, titled ‘Advances in Quantitative Bioimaging’, proposes an overview of the latest advancements in quantitative bioimaging techniques and their wide-ranging applications. The articles cover various topics, including modern imaging methods that enable visualization on a nanoscale, such as super-resolution microscopy and single-particle analysis. These techniques offer unparalleled insights into complex molecular structures and dynamic cellular processes *in situ*, such as mapping nuclear pore proteins or tracking single histone deposition events throughout the cell cycle. The articles presented in this edition showcase cutting-edge quantitative imaging techniques coupled with advanced computational analysis capable of precisely measuring biological structures and processes. Examples range from correlating calcium release events to underlying protein organization in heart cells to pioneering tools for categorizing changes in microglia morphology under various conditions. This editorial highlights how these advancements are revolutionizing our understanding of living systems, while acknowledging challenges that must be addressed to fully exploit the potential of these emerging technologies, such as improving molecular probes, algorithms and correlation protocols.

## 1. Introduction

Exploring life's intricate mechanisms calls for an up-close examination of biological structures from every vantage point, from molecules to cells to tissues. Recent progress in quantitative bioimaging technologies has empowered us to visualize complex molecular architectures, track individual proteins, compare cellular dynamics with nanoscale precision and single-molecule resolution. This leap forward holds significant potential for driving our understanding into uncharted territory by offering fresh mechanistic insights into living systems. In this special feature issue of *Open Biology* titled ‘Advances in Quantitative Bioimaging’, we present breakthrough research demonstrating how innovative imaging modalities combined with new computational analysis methods are providing an intimate glimpse into heretofore unseen molecular aspects of dynamic cellular processes.

## 2. Contributions

This special edition features articles highlighting the ground-breaking applications of quantitative bioimaging techniques in various areas of biology. A review by Mendes *et al*. discusses the power of combining super-resolution microscopy (SRM) with single-particle analysis (SPA). Several primary research articles in this special edition leverage advanced imaging methods to shed light on important cellular processes: Hurley *et al*. explore the relationship between calcium signalling events and the structural organization of calcium channels in cardiac muscle cells; Lando *et al*. use single-particle tracking to understand histone CENP-A molecule deposition in fission yeast; Ragaller *et al*. propose novel smart probes to measure membrane properties, which are crucial for understanding cellular processes. On the computational front, Martinez *et al*. have developed machine learning-based tools to characterize morphological alterations and behavioural shifts in microglia during neuroinflammation.

Mendes *et al*., in their review entitled ‘Mapping molecular complexes with super-resolution microscopy and single-particle analysis’, discuss the combined power of SRM and SPA [1]. SRM's ability to identify nanoscale biological structures is generally limited by the detected labelling density. SPA allows SRM to transcend these limitations by combining a series of images for similar objects to generate higher-resolution models. Their study spotlights the effectiveness of SRM–SPA to map intricate complexes like nuclear pores, endocytic machinery, viruses, cilia and centrioles. However, challenges remain including bias from initial templates and limited dynamic capture. Nevertheless, the recent adoption of template-free algorithms, along with particle classification and live-cell imaging correlation, are now addressing some of these issues.

Miriam E. Hurley and her colleagues in their paper ‘Correlative super-resolution analysis of cardiac calcium sparks and their molecular origins in health and disease' have successfully linked calcium release events, referred to as ‘sparks’, with the nanoscale arrangement of calcium channels known as ryanodine receptors (RyR2) in rat heart cells [2]. Using a combination of total internal reflection fluorescence microscopy and DNA-point accumulation for nanoscale topography super-resolution imaging methods, they compared spontaneous calcium sparks with underlying RyR2 receptor maps on a spark-per-spark basis within cardiomyocytes. The results showed that healthy cells facilitate sparks through combinations of RyR2 clusters, whereas failing heart cells display greater inconsistency in spark mass despite involving more RyR2 channels. This experimental evidence indicates that the clustering patterns of RyR2 and the functional coupling between clusters are crucial for generating sparks, and suggests potential structural–functional relationships that are hidden by traditional methodology.

The article ‘*In vivo* assessment of mechanical properties during axolotl development and regeneration using confocal Brillouin microscopy’ by Riquelme-Guzmán *et al*. used confocal Brillouin microscopy to uncover tissue mechanical changes *in vivo* throughout development and regeneration in the highly regenerative axolotl, with a particular focus on limb and digit cartilage [3]. Probing tissue mechanical properties has become relevant for a better understanding of a plethora of biological processes. Overall, the work emphasises the potential of confocal Brillouin microscopy to address relevant questions from a mechanical perspective in the growing field of regenerative biology.

In the article titled ‘Characterization of microglia behaviour in healthy and pathological conditions with image analysis tools’ by Martinez *et al*., the authors used image analysis tools to examine the morphology and phagocytic behaviour of microglia in healthy and pathological conditions *in vitro* and in brain tissue [4]. The authors observed microglia engulfing cell debris after excitotoxicity and inflammation using time-lapse imaging of neuron-glia cultures. They classified microglia into seven categories based on morphometric features. Machine learning helped cluster cells into these classes with 82% accuracy. In control cultures, round and hypertrophic microglia were predominant. Excitotoxicity modestly increased hypertrophic microglia while adding inflammation significantly enriched inflamed subtypes. For tissue validation, the segmentation pipeline identified ramified and amoeboid microglia distributions in non-ischaemic versus peri-infarct regions. These computational tools allowed for quantitatively characterizing microglia heterogeneity and response to neuroinflammation with subcellular resolution.

The research presented in this special issue has important implications for various fields. For instance, Ragaller *et al*., in their work ‘Dissecting the mechanisms of environment sensitivity of smart probes for quantitative assessment of membrane properties', present smart probes—Pro12A, NR12S and NR12A—to assess membrane properties [5]. In their study, the authors investigated the mechanisms underlying the probes' sensitivity to the local environment. They used model membrane systems with defined lipid compositions, spectral imaging, molecular dynamics simulations and time-dependent fluorescence shift analysis to compare the probes' ability to detect differences in lipid saturation, double bond position/configuration, headgroup and cholesterol content. While all three probes can differentiate between liquid-ordered and disordered phases, they exhibit distinct sensitivities to various membrane properties based on their orientation in the bilayer and relaxation dynamics. Pro12A is best suited to sense cholesterol content and fluidity, NR12S outperforms in detecting saturation and headgroup changes, and NR12A is most sensitive to double bond position/configuration. This study demonstrates the selective application or combination of these probes to quantitatively assess different aspects of membrane biophysics relevant to cellular processes and disease states. It provides a guide for choosing optimal smart probes tailored to the membrane feature under investigation.

## 3. Conclusion

The studies presented here highlight scientific progress made possible by advanced quantitative bioimaging techniques and image analysis. These tools allow us to better understand the intricate layers of cellular functions and expand our perception of how living systems operate across different scales. Over time, these advancements will help bridge the gap between experimental observation and theoretical vision, progressively tackling the formidable complexity intrinsic to biology.

## Data Availability

This article does not contain any additional data.
